# Supplementary material for: Intrinsic multiplication rate variation and plasticity of human blood stage malaria parasites
Source: Commun Biol. 2020 Oct 28;3:624. doi: 10.1038/s42003-020-01349-7 (PMC7595149; doi:10.1038/s42003-020-01349-7)
Supplement: Supplementary file 3 — Description of Additional Supplementary Files [file 42003_2020_1349_MOESM3_ESM.docx]

**Description of Additional Supplementary Files**

File name: Supplementary Data 1

Description: Raw data from exponential multiplication rate assays conducted over 6 days (isolates being sampled from maintenance culture from day 25, 77, and 153). Parasite genome copy numbers were derived by qPCR of the target single-copy gene locus PF3D7_1031000. Multiplication rate was estimated by logistic regression using all experimental replicate data following technical quality control. The triplicate experimental replicates are shown underneath each isolate identifier.
